# Warm Vapors of Formalin as Disinfecting Agents

**Published:** 1918-12

**Authors:** 


**Warm Vapors of Formalin as Disinfecting Agents.** By MM. Guillaume-Louis and Rousseau. *Bulletin de la Société de Chirurgie de Paris.* May 28, 1918.

Formalin vapors are generally obtained in heating trioxymethylen, when needed for disinfection. But sometimes this has proved incomplete. Spores of bacteria seem to resist this vaporous action.

MM. Guillaume-Louis and Rousseau have therefore introduced a new method of sterilization at the surgical auto-ambulance where they have experimented.

To begin with, all instruments undergo what they call " presterilization ". After every operation, the instruments and gloves are put into a basin containing water and 15 gr. of borax, and 5 cc. of a formalin solution at 40 per cent. The whole is heated at 80° C., and this temperature is kept on for about 10 minutes. Then the instruments and gloves are dipped into a borax solution, brushed carefully, and dried. They are finally sterilized, and a double gauze is spread on metal trays. On this gauze are pulverized a few cc. of a formalin solution at 40 per cent., previously neutralized by q. s. of soda, as it always contains small quantities of acetic and formic acids that would damage the steel. Instruments are laid on this gauze, and covered with another sheet of gauze. The trays are then left for 3/4 of an hour at 70 or 80° C. in a dry and warm autoclave. After that time, the formalin vapors are pumped out of the autoclave, aseptic air takes their place, and the instruments are then ready for use.

The efficacity of this disinfection is tested in the following way : small pieces of compresses are soaked in virulent cultures of different bacteria, and then deposited on the trays, beneath and above the instruments. After the sterilization these pieces are introduced into tubes containing Martin's broth. They are maintained there 48 hours at 37° C., and then tubes of Veillon's gelose are inoculated with this culture. Seven experiments of this kind were repeated without success. No bacteria ever developed; the broth always proved to be sterile. The experiments were carried out with the following bacteria : Subtilis, Perfringens, Sporogenes, Putrificus, Vibrio septicus, all of these containing a certain amount of spores. In each experiment, one of the infected compresses was left in the open air, acting as control.

This method, according to the author, presents the following advantages :

1) The temperature of 80° C. does not soften the steel, nor does it spoil the cutting edges of the bistouries or scissors.

2) It proves more active than the heating of trioxymethylen. This substance releases formalin vapors, but only in limited quantities, when it is heated in a closed space. This is due to the strong polymerization power of these vapors. If the temperature rises, the tension of the vapors increases, and polymerization becomes more active. Therefore, the atmosphere enclosed in the autoclave never contains a quantity of disinfecting vapors sufficient to destroy the spores of bacteria.

Polymerization does not interfere when formalin vapors are obtained by heating a formalin solution at 40 per cent.

